# Unravelling Antigenic Cross-Reactions toward the World of Coronaviruses: Extent of the Stability of Shared Epitopes and SARS-CoV-2 Anti-Spike Cross-Neutralizing Antibodies

**DOI:** 10.3390/pathogens12050713

**Published:** 2023-05-13

**Authors:** Christian A. Devaux, Jacques Fantini

**Affiliations:** 1 Laboratory Microbes Evolution Phylogeny and Infection (MEPHI), Aix-Marseille Université, IRD, APHM Institut Hospitalo-Universitaire—Méditerranée Infection, 13005 Marseille, France; 2 Centre National de la Recherche Scientifique (CNRS-SNC5039), 13009 Marseille, France; 3 Aix-Marseille Université, INSERM UMR_S 1072, 13015 Marseille, France

**Keywords:** SARS-CoV-2, coronaviruses, immune response, cross-reaction, spike, ACE2, zoonoses, one health

## Abstract

The human immune repertoire retains the molecular memory of a very great diversity of target antigens (epitopes) and can recall this upon a second encounter with epitopes against which it has previously been primed. Although genetically diverse, proteins of coronaviruses exhibit sufficient conservation to lead to antigenic cross-reactions. In this review, our goal is to question whether pre-existing immunity against seasonal human coronaviruses (HCoVs) or exposure to animal CoVs has influenced the susceptibility of human populations to SARS-CoV-2 and/or had an impact upon the physiopathological outcome of COVID-19. With the hindsight that we now have regarding COVID-19, we conclude that although antigenic cross-reactions between different coronaviruses exist, cross-reactive antibody levels (titers) do not necessarily reflect on memory B cell frequencies and are not always directed against epitopes which confer cross-protection against SARS-CoV-2. Moreover, the immunological memory of these infections is short-term and occurs in only a small percentage of the population. Thus, in contrast to what might be observed in terms of cross-protection at the level of a single individual recently exposed to circulating coronaviruses, a pre-existing immunity against HCoVs or other CoVs can only have a very minor impact on SARS-CoV-2 circulation at the level of human populations.

## 1. Diversity and Interspecies Circulation of Coronaviruses

**Figure 1:**
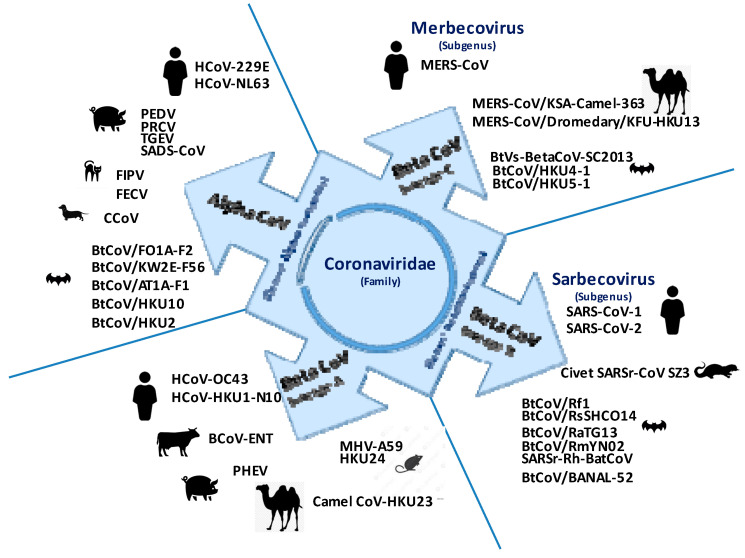
Schematic representation of phylogenetic clustering of representative strains belonging to the alpha-coronaviruses and beta-coronaviruses (lineages A, B, and C) genera in humans and animals. The CoVs are divided into four distinct phylogenetic groups (CoV genera), defined as α and β known to infect mammals, while γ and δ (not shown for γ and δ) infect both mammals and birds [1]. This taxonomic nomenclature replaced the former groups 1, 2, and 3 (http://ictvonline.org/proposals/2008.085-122V.v4.Coronaviridae.pdf (accessed on 3 May 2023)). Of course, the aim here is not to show an exhaustive diagram of all coronaviruses encountered in humans and animals, but simply to highlight their most known representatives. For more details see references [2,14] and https://ictv.global/taxonomy (accessed on 3 May 2023).

Coronaviruses (CoVs) are a group of large single-stranded ribonucleic acid (RNA) viruses, belonging to the order Nidovirales, family Coronaviridae, and are classified into four distinct phylogenetic groups (or genera), based on differences in protein sequences: alpha and beta, (α and β are known to infect mammals) and delta and gamma (γ and δ are known to infect both mammals and birds) CoVs genera, and their subgenera [1,2,3]. These four CoVs genera are predicted to have diverged millions of years ago [4], and the circulation of these viruses in different animal hosts has resulted in a myriad of recombination events [5,6]. To date, seven types of coronaviruses have been found to infect humans. They include four endemic genotypes (HCoV-229E, HCoV-NL63, HCoV-OC43, and HCoV-HKU1) which are classified as low-pathogenic human coronaviruses, as they usually only cause mild upper respiratory tract infections although some of them can cause severe infections in infants and the elderly [7,8]. In contrast, three genotypes have been found that can cause severe acute respiratory diseases, including the Middle East respiratory syndrome coronavirus (MERS-CoV) [9] and the severe acute respiratory syndrome coronaviruses (SARS-CoV1 and SARS-CoV-2) [10,11,12,13], classified as highly pathogenic human coronaviruses (Figure 1).

**Figure 2:**
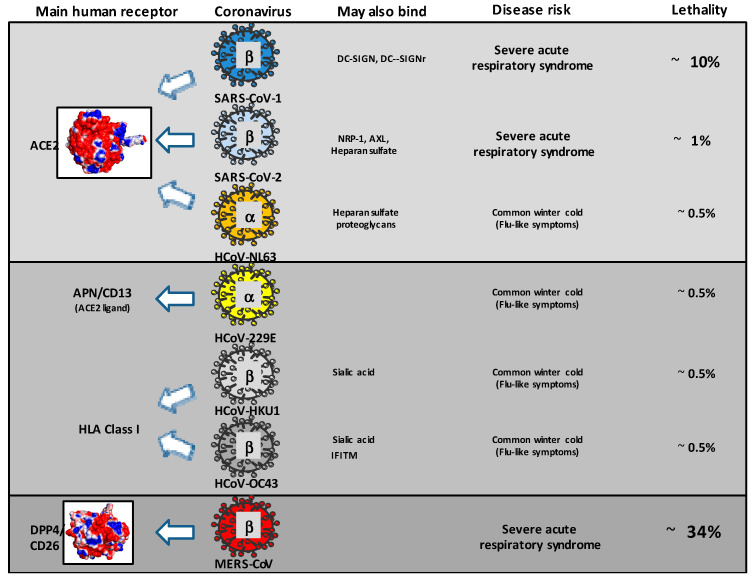
Tropism of coronaviruses infecting humans. The coronaviruses known to infect humans are classified according to their tropism (main human receptor). Their classification with respect to the Alphacoronavirus genus (α) and Betacoronavirus genus (β) is indicated. The representation also indicates the possible severity and lethality of the disease. Although the lethality of SARS-CoV-2 is relatively low compared to other Sarbecovirus and Merbecovirus, it was responsible for a higher number of deaths due to its pandemic spread.

The first HCoVs described in the 1960s as causative agents of the common winter cold were HCoV-229E (Alphacoronavirus/Duvinacovirus) and HCoV-OC43 (Betacoronavirus lineage 2a/Embecovirus). In 2003, the “human” coronaviruses gained in notoriety with the emergence of the highly pathogenic SARS-CoV-1 (Betacoronavirus lineage 2b/Sarbecovirus), causing a severe acute respiratory syndrome with a case fatality rate of 9.6% [10]. The human angiotensin converting enzyme 2 (hACE2) was found to be the functional receptor for SARS-CoV-1 [15]. Within the next couple of years, both the HCoV-NL63 (Alphacoronavirus lineage 1b/Setracovirus), that also uses ACE2 as a receptor, and HCoV-HKU1 (Betacoronavirus lineage 2a/Embecovirus), that uses aminopeptidase N/CD13 as a receptor, were discovered in human patient samples [16]. Notably, the spike from HCoV-NL63 not only binds to hACE2 but ACE2 from horses as well [17]. HCoV-OC43 emerged through a single zoonotic introduction, using 9-O-acetylated sialic acid as a receptor and, following introduction to the human population, the viral hemagglutinin-esterase protein-mediated receptor binding was ultimately lost. This took place through the progressive accumulation of mutations in the HE lectin domain to downregulate the receptor-destroying activity likely to meet the specific requirements for optimal replication in human airways, a mechanism also observed with HCoV-HKU1 [18]. HCoV-NL63 diverged from HCoV-229E around the 11th century, dated using the Bayesian coalescent approach, and appears to have resulted from recombination between an ancestral CoV-NL63-like virus and an ancestral CoV-229E [19,20]. To date, the case fatality rate of the four HCoVs is considered to be between 0.5% and 1.5% [21,22,23], most frequently around 0.5%. The emergence of MERS-CoV (Betacoronavirus lineage 2c/Merbecovirus). originating from a recent zoonotic event, was reported in 2012 and the epidemic was characterized by a surprising and extremely high case fatality rate of 34.7% [9]. The dipeptidyl peptidase (DPP4)/CD26 was identified as the MERS-CoV receptor [24,25]. The latest and seventh human coronavirus, SARS-CoV-2 (Betacoronavirus lineage 2b/Sarbecovirus), emerged in China in 2019 and showed 79.5% nucleotide identity with SARS-CoV-1 [26]. The cell entry of SARS-CoV-2 depends on the ACE2 receptor and the TMPRSS2 protease [27,28]. The emergence cause of SARS-CoV-2 is still quite heavily debated [29]. The case fatality rate of SARS-CoV-2 was not particularly high, around 1%, but the virus spread so rapidly worldwide that it became responsible for several million human deaths.) (Figure 2).

**Figure 3:**
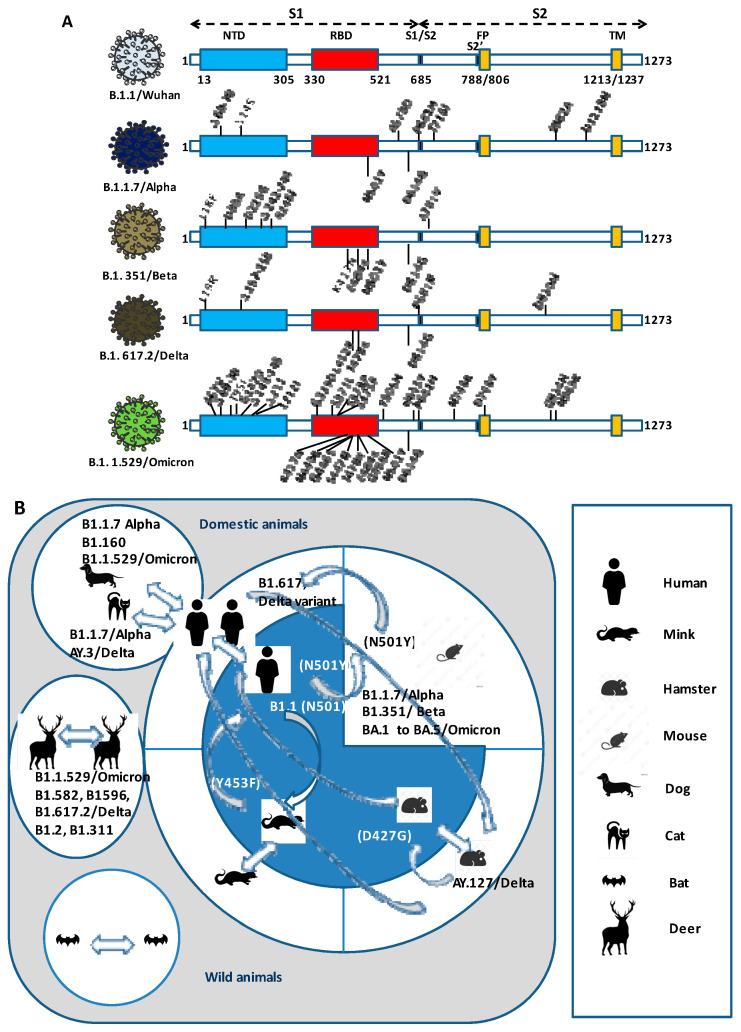
Intra- and interspecies circulation of SARS-CoV-2 lineages/sub-lineages. (**A**) Schematic representation of SARS-CoV-2 variants spike Alpha, Beta, Delta and Omicron lineages. The spike (S) protein (1273 amino acids) is comprised of an N-terminal subunit (S1) that mediates the receptor binding and a C-terminal subunit (S2) responsible for virus-cell membrane fusion. NTD: N-terminal domain; RBD: receptor-binding domain; FP: fusion peptide; TM: single-span transmembrane domain. D: deletion. Substitutions with respect to the original SARS-CoV-2 Wuhan, are indicated. (**B**) SARS-CoV-2 circulation in animal species. According to the reference site Vetmeduni/Complexity Science Hub, Vienna (https://vis.csh.ac.at/sars-ani/ (accessed on 3 May 2023)) that reported an overview of SARS-CoV-2 events in animals from January 2020 to December 2022 (616 outbreaks of SARS-CoV-2 had been reported in 31 animal species with a 2.8% fatality rate occurring in 39 countries).

In addition, since the start of the pandemic, we witnessed the rapid replacement of one SARS-CoV-2 lineage by another. This contrasts with the early claims that the genetic diversity of SARS-CoV-2 was extremely low [30,31]. Genetic diversity is clearly a major determinant of vaccine efficacy. The RNA-dependent RNA polymerase gene (RdRp known as nsp12) of coronaviruses is known to be error-prone, and it has been reported that SARS-CoV-2 with a P_314_L substitution in the *nsp12* increases errors by a factor of three [32] and a P_203_L substitution in the proofreading subunit nsp14 increases errors by a factor of two [33]. Notably, one recent report [34] established that the fidelity of SARS-CoV-2 nsp12, along with its co-factors nsp7 and nsp8 and in the absence of the nsp14, is 10^−1^–10^−3^, compared to a fidelity of 10^−6^–10^−7^ for other coronaviruses. This is most likely due to critical mutations in nsp12 and nsp14. Thus, it is it not surprising that viral variants rapidly arise during the in vivo passage of the virus. Repeated intra- and interspecies transmission of SARS-CoV-2 presents the potential for acceleration of genetic drift and a possible source of novel strain emergence. This was demonstrated by the reverse zoonosis of SARS-CoV-2 from humans to mink, followed by selection in mink and zoonotic transmission back to humans [35] (Figure 3).

The first encounter with a virus induces a primary immune response with a proliferation of naive antigen-reactive B lymphocytes that undergo immunoglobulin (Ig) class switching (e.g., IgM to IgG) leading to mature plasmocytes secreting short half-life immunoglobulin in plasma (e.g., a half-life of between seven and 21 days for IgG), while long-lived memory B cells convey the potential to engage in a more efficient anamnestic secondary immune response if the same virus, or an antigenically related virus, is encountered later in life. Each viral infection is expected to prompt our bodies to make potent Ig responses including neutralizing antibodies aimed at blocking the virus from infecting cells. Since the beginning of the SARS-CoV-2 pandemic and with the ultimate goal of developing a vaccine against SARS-CoV-2, immunologists have worked feverishly to determine what immunity to SARS-CoV-2 could look like and whether or not pre-pandemic cross-reactive immunity may have contributed towards reducing the percentage of symptomatic COVID-19 in people exposed to SARS-CoV-2. This consists of studying whether individuals who had been previously infected with another coronavirus share any epitopes with SARS-CoV-2 in the patchwork of potential epitopes inducing neutralizing antibodies. Here, we review the evidence indicating that the immune system can recall preexisting memory B cells specific from their distinctive histories of infections to respond to SARS-CoV-2, and we discuss to what extent preexisting immunity against seasonal human coronavirus (HCoV) or exposure to animal CoV could have influenced the transmission of SARS-CoV-2 in humans and/or the severity of the COVID-19.

## 2. Evidence of Cross-Reactivity towards HCoV, MERS-CoV, SARS-CoV-1, and SARS-CoV-2

Could a repertoire of anti-SARS-CoV-2 immunoglobulins pre-exist a primary infection with this virus in some individuals who would have been immunized following encounters with other “human” coronaviruses?

**Figure 4:**
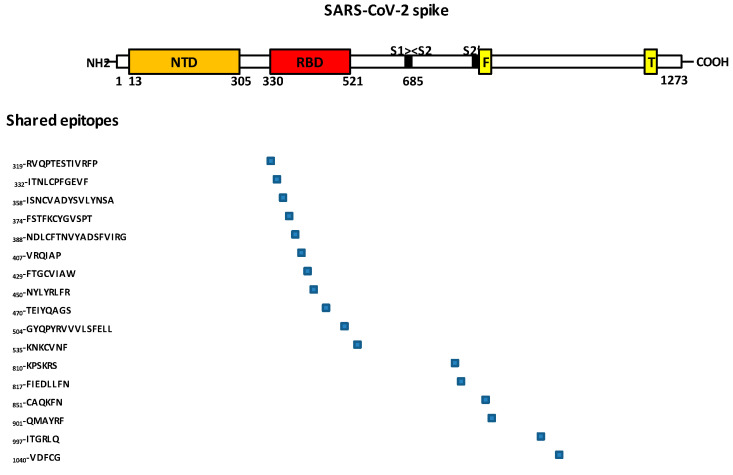
Shared epitopes between HCoVs, MERS-CoV and SARS-CoVs spike proteins. The alignment of HCoV-HKU1, HCoV-NL63, HCoV-229E, HCoV-OC43, MERS-CoV, and SARS-CoV-1 spike (S) protein sequences with the S protein sequence of SARS-CoV-2 (Ref: UniProtKB/Swiss-Prot:P0DTC2.1) suggests possible cross-reactive immunity against several peptides found in the S protein sequence of SARS-CoV-2 (blue boxes) if the host immune repertoire has previously been primed with a human coronavirus other than SARS-CoV-2. S1: spike N-terminal subunit; S2: spike C-terminal subunit; NTD: N-terminal domain; RBD: receptor-binding domain; F: fusion peptide; T: single-span transmembrane domain.

Low-pathogenic seasonal endemic HCoV are continuously circulating among the global population and are assumed to be responsible for about 5% of all acute respiratory tract infections worldwide. They are more prevalent in young children (under five years old), the population which is the least affected by severe COVID-19 associated with the early circulating SARS-CoV-2 lineages [36,37]. Immunological memory after infection with HCoV may potentially contribute to short lasting cross-protection against SARS-CoV-2. A recent comparison of the seven coronaviruses infecting human (the four HCoVs, as well as MERS-CoV and the two SARS-CoVs) indicated that overall amino acid sequence identity (pairwise gaps excluded) was low across these viruses, with 23.9% for the envelope (E), 36.2% for the membrane (M), 36.3% for nucleocapsid (N), and 29.5% for the spike (S), while it was 93% for E and 78% for S between the two SARS-CoVs [38]. This had already been reported decades ago, when research teams were searching for shared epitopes between HCoV and SARS-CoV-1. They reported that the probability that proteins from different strains of HCoV share epitopes with Sarbecoviruses was higher with the viral nucleocapsid N phosphoprotein, which is more highly conserved compared to S (S) [39]. However, antibody levels to N do not correlate with the neutralizing activity [40] which is mainly conferred by anti-S immunity. Strong cross-reactivity has been observed between SARS-CoV-1-positive plasma and the SARS-CoV-2 N protein [41,42]. The spike protein of HCoV-HKU1, HCoV-NL63, HCoV-229E, and SARS-CoV-2, analyzed by a pairwise similarity plot, showed that HCoV-HKU1 had the highest similarity in all spike regions to the SARS-CoV-2 spike, compared with HCoV-229E and HCoV-NL63 [43]. SARS-CoV-1 was reported to share epitopes with HCoV-OC43, and weak cross-reactions were also found with HCoV-229E [44,45,46]. Che and colleagues [45] found that sera from SARS-CoV-1 showed a several fold increase in antibody titers against HCoV-229E and HCoV-OC43 compared to controls. Hu and colleagues [47] reported that preexisting antibodies to HCoV-OC43 in healthy people were positively correlated with an immune response against the receptor binding domain (RBD) found in the S protein of SARS-CoV-2 and neutralization antibodies to SARS-CoV-2 in people who had been vaccinated against SARS-CoV-2. The alignment of these coronaviruses S protein sequences suggested possible cross-reactive immunity against peptides _319_-RVQPTESTIVRFP, _332_-ITNLCPFGEVF, _358_-ISNCVADYSVLYNSA, _374_-FSTFKCYGVSPT, _388_-NDLCFTNVYADSFVIRG, _407_-VRQIAP, _429_-FTGCVIAW, _450_-NYLYRLFR, _470_-TEIYQAGS, _504_-GYQPYRVVVLSFELL, and _535_-KNKCVNF, some of which (e.g., _407_-VRQIAP) had been previously identified as SARS-CoV-2 spike protein S1 subunit (S1) linear B cell epitopes [48,49]. Ng and colleagues [50] reported that in a cohort of 350 SARS-CoV-2–uninfected individuals, a small proportion who had had a confirmed HCoV infection had circulating antibodies that could cross-react with the SARS-CoV-2 spike protein S2 subunit (S2), S2 exhibiting a higher degree of homology among coronaviruses than S1. These authors mapped several linear B cell epitopes derived from the S2 of SARS-CoV-2 at positions _810_-KPSKRS, _817_-FIEDLLFN, _851_-CAQKFN, _901_-QMAYRF, _997_-ITGRLQ, and _1040_-VDFCG, fairly well conserved with HCoV-229E, HCoV-NL63, HCoV-OC43 and HCoV-HKU1 (Figure 4).

Cross-reactivity was also investigated within different lineage of SARS-CoV-2. Using sera from individuals recovering from infection with either the B1 (D614G) or B1.617.2 (Delta) lineages of SARS-CoV-2, Rak and colleagues [51] analyzed the anti-N protein antibodies and found that they are highly cross-reactive among SARS-CoV-2 lineages, and that the most immunogenic epitopes within this protein are not under selective pressure, since these epitopes are conserved between the ancestral B.1 virus and the B.1.351 (Beta), P1 (Gamma), B.1.617.2 (Delta), and B.1.1.529 (Omicron) lineages. Using SARS-CoV-2 enzyme immunoassays and microneutralisation assays, To and colleagues [52] found a seropositivity rate of 2.73% (53 of 1938 samples) in sera from individuals who had probably never been exposed to SARS-CoV-2 (serum samples collected before 2019). SARS-CoV-2-reactive antibodies were detected in unexposed individuals who were seropositive for HCoV-OC43 and HCoV-NL63.

Although there have not been many people infected with SARS-CoV-1 or MERS-CoV, some studies suggest antigenic cross-reaction with SARS-CoV-2 [53,54]. In contrast, Maani and colleagues [55] reported a lack of cross-protection between MERS-CoV and SARS-CoV-2. Another study reported the probable lack of a cross immune response between MERS-CoV and HCoV [56]. Indeed, the probability that the circulation of MERS-CoV or SARS-CoV-2 could have modified the dynamics of the SARS-CoV-2 pandemic is zero. By probing a phage-displayed antigen library of 12,924 peptides corresponding to sequences from the four HCoV, MERS-CoV, SARS-CoV-1, and SARS-CoV-2 and 49 different animal CoVs with antibodies from pre-pandemic individuals (260 samples collected between 2013 and 2016) and patients who had recovered from COVID-19 (269 samples collected in 2020), Klompus and colleagues [57] found that unexposed individuals showed an abundant antibody responses against all seasonal HCoVs (e.g., 88% bound peptides of HCoV-NL63, 87% bound peptides of HCoV-HKU1), while sera from patients who had recovered from COVID-19 bound significantly more peptides of SARS-CoV-2 but also cross-reacted with peptides from SARS-CoV, MERS-CoV, HCoV-OC43, and HCoV-HKU1. They also characterized shared epitopes of SARS-CoV-2, HCoV-OC43, HCoV-HKU1, and several animal CoVs (e.g., a peptide recognized by the A7 mAb and derived from the SARS-CoV-2 spike at position 748 was found to be similar to a peptide that is part of a bat SARS-like CoV S protein), and confirmed the existence of the conserved _815_-RSFIEDLLFNK sequence in S2, shared between SARS-CoV-2 and HCoV and previously reported by Shrock and colleagues [49]. In addition to this epitope at position 815 conserved with the four HCoVs, Shrock and colleagues identified another linear epitope derived from the S protein of SARS-CoV-2 in S2 (_1148_-FKEELDKYF), only shared with HCoV-OC43 and weakly conserved in HCoV-HKU1. Song and colleagues [58] described a protective neutralizing antibody named mAb CC40.8, targeting the bottom of the S2 of HCoV-HKU1 and SARS-CoV-2. More recently, by profiling the antibody response in COVID-19 naive individuals with a diverse clinical history (including cardiovascular, neurological, or oncological diseases), Jaago and colleagues identified 15 highly antigenic epitopes on the SARS-CoV-2 spike protein (ten in S1, including three in the S1 N-terminal domain, NTD, four in the S1 RBD, and five in S2) that showed cross-reactivity with antigens of seasonal HCoV, as well as persistent, latent or chronic infections from common human viruses, such as cytomegalovirus and Epstein–Barr virus [59]. Notably, it was reported that the BNT162b2 (SARS-CoV-2/Pfizer) vaccination increased the antibody neutralizing response against HCoV-HKU1 [60,61], whereas the mRNA-1273 (SARS-CoV-2/Moderna) vaccination increased antibody neutralizing response against HCoV-NL63, and the AZD1222 (SARS-CoV-2/Astrazeneca) vaccination increased antibody neutralization against HCoV-229E [43]. The molecular rationale for such specific SARS-CoV-2/HCoV cross-reactions of differently engineered anti-S vaccine preparations remains to be explored and could result from different modifications made to the sequence of the S protein to stabilize its conformation (e.g., substitutions K_986_P and V_987_P). However, it has been observed that, while there was a small boost in antibodies towards HCoVs during SARS-CoV-2 infection, this was not associated with protection and, conversely, prior infection with an HCoV apparently did not protect against SARS-CoV-2 [62,63].

## 3. Evidence for Cross-Reactivity towards Animal CoVs, HCoVs, and Human CoVs including SARS-CoV-2

In the same way that some individuals can be partly immunized against SARS-CoV-2 following stochastic encounters with other “human” coronaviruses, could a repertoire of antibodies able to cross-react with SARS-CoV-2 pre-exist a primary infection with this virus in some individuals who would have developed an immune response following encounters with “animal” coronaviruses?

If this has ever happened, it should obviously be a much rarer event than cross-immunity due to HCoV infection, because it requires humans to be a susceptible host for these specific animal viruses, something which has so far not particularly attracted the attention of the viral surveillance institutions. It is particularly difficult to estimate the frequency of human exposure to animal coronaviruses and potential zoonosis of animal coronaviruses from animals to humans. Up to now, it can be considered that transmission of animal coronaviruses to humans is extremely rare. Using a multiprobe for coronaviruses, a retrospective study of 200 human nasal swab samples collected by the Arkansas Department of Health in 2010 for influenza diagnosis, found FCoV-like sequences in three samples, representing possible evidence of interspecies transmission or a new human strain [64]. Recently, two independent studies have reported canine coronavirus (CCoV; alphacoronavirus) infection in humans who showed symptoms of mild fever and pneumonia, in Malaysia and the USA [65,66]. A study that addresses the subject of animal respiratory viruses transmitted to humans in individuals with a high frequency of animal exposure mainly highlight that enteroviruses, rhinoviruses and influenza viruses are the most often detected, while coronaviruses were not found [67]. It has also been suggested that porcine deltacoronaviruses, which can infect human cell lines, could be transmitted to humans [68,69]. In fact, more than two decades ago, a virus closely related to the porcine epidemic diarrhea virus (PEDV) was found in an 8-month-old boy suffering from pneumonia in the Netherlands in 1988, and this virus was also detected in four of 139 individuals (3%) who were suffering from respiratory illness with unknown etiology [70]. PEDV, which belongs to the alphacoronaviruses alongside HCoV-229E and HCoV-NL63, was used to evaluate the transboundary risk of PEDV-contaminated feed ingredients. This experiment suggested the ability of the virus to survive in long distance shipments and the possibility of coming into contact with humans [71]. The same is probably true for a large number of known and unknown animal coronaviruses, some of which are potentially able to infect humans, and which share epitopes with HCoV, Merbecoviruses, or Sarbecoviruses. This was observed with the swine acute diarrhea syndrome coronavirus (SADS-CoV), related to the bat BtCoV/HKU2, which replicates efficiently in primary human lung cells, human intestinal cells, and human carcinoma cell lines [72], suggesting that its transmission to humans is likely to be possible. The detection of Alphacoronaviruses and Gammacoronaviruses in seafood has also been reported [73,74], which could make human infection possible through eating raw fish. The fear associated with this concept has triggered a decrease in annual demand and sales of fresh and frozen seafood in many countries and a 30–40% decrease in exports during the early phase of the SARS-CoV-2 pandemic [75].

Evidence for cross-reactivity between animal and human coronaviruses was reported several decades ago with the description of shared epitopes between the HCoV-229E and coronaviruses from domestic animal such as feline infectious peritonitis virus (FIPV), porcine transmissible gastroenteritis virus (TGEV), and canine coronavirus (CCoV) [76,77], as well as between the HCoV-OC43 and bovine CoV or mouse hepatitis coronavirus (MHV) [78]. It has been reported [44] that polyclonal antibodies from antigenic group 1 coronaviruses, including FIPV, and TGEV, reacted with SARS-CoV-1-infected cells. Sun and Meng [39] found that the N protein of SARS-CoV-1 reacted with polyclonal antisera of known antigenic group 1 coronaviruses TGEV, FIPV, and CCoV, indicating that the N protein of SARS-CoV-1 shares common antigenic epitope(s) with those viruses. However, this SARS-CoV-1 N protein did not cross-react with polyclonal antisera from antigenic group 2 animal coronaviruses, including the porcine hemagglutinating encephalomyelitis virus (HEV) and bovine coronavirus (BCoV), or group 3 animal coronaviruses, including turkey coronavirus (TCoV) and avian infectious bronchitis virus (IBV). Shared epitopes were also found between the N protein of SARS-CoV-1 amino acids 120 to 208 and both porcine TGEV and porcine respiratory CoV (PRCV) [79]. Although cross-reactivity epitopes in the N protein were found within SARS-CoV-1 and bat BtCoV/HKU3N and BtCoV/279N (belonging to the same subgroup as SARS-CoV-1), as well as within the MERS-CoV and bat BtCoV HKU5.5N subgroup, there was an absence of cross-neutralization [80]. Recombination analysis also suggested the possibility of recombinant events between different sources of CoV (e.g., bat CoV and PEDV), suggesting frequent gene transfers which may be the result of the cross-species transmission of these CoVs [19,81]. Recombination in CoVs was first recognized between different strains of murine hepatitis virus (MHV) and subsequently in other animal CoVs [82,83], including civet SARSr-CoV SZ3 [84], as well human CoVs such as HCoV-HKU1 [85] and SARS-CoV-2 [86,87].

Notably, the closest sequences to SARS-CoV-2 characterized in wild animals were found in bats. One of the first papers reporting on SARS-CoV-2 was entitled “Pneumonia outbreak associated with a new coronavirus of probable bat origin”, and the authors reported 96% sequence identity between SARS-CoV-2 and the bat coronavirus RaTG13 sequence obtained from a Rhinolophus affinis bat [26]. Although bats are very often carriers of coronaviruses [14,88,89,90,91], some of which are very similar to SARS-CoV-2, there is no indication that a bat Sarbecovirus (any more than a pangolin Sarbecovirus, as has long been suggested in the scientific literature), was the cause of pandemic SARS-CoV-2 in humans [92]. However, efforts were focused on bats in an attempt to characterize an ancestral form of SARS-CoV-2 in animals and, since the identification of RaTG13 sequence, several batCoV sequences, including the RacCS203 from a Rhinolophus acuminatus bat, the RmYN02 batCoV sequence from a Rhinolophus malayanus bat, and the RshSTT182 and RshSTT200 from Rhinolophus shameli bats, sharing between 95.86% and 92.6% similarity with SARS-CoV-2, respectively, have been reported [93,94,95]. A new bat Sarbecovirus (namely BANAL-52 virus) isolated from a Rhinolophus malayanus bat in Laos was reported to exhibit 96.8% identity with SARS-CoV-2 throughout the length of the genome, and experiments on infection with recombinant viruses suggested the same potential for infecting humans through hACE2 as early strains of SARS-CoV-2 [96]. No systematic survey of bat-borne CoVs infection in humans does exist. Although there are currently no known reports of people carrying a CoV from a bat origin without falling ill, if some people living in a bat ecosystem could get infected by such viruses, cross-reaction with HCoV, Sarbecoviruses, or Merbecoviruses remains possible.

## 4. Epitopes in SARS-CoV-2 RBD That Binds ACE2 and Are Related to Virus Neutralization

The bibliographic data mentioned above clearly indicate that individual contacts with coronaviruses regularly circulating in humans (such as HCoV), and even contacts with coronaviruses known to circulate in animals (such as FCoV, CCoV or PEDV), may have led to the creation of the conditions for cross-immunity with SARS-CoV-2. However, what makes sense is not the cross-reaction itself, but the cross-reaction which allows neutralization of the virus and the ability of these neutralizing antibodies to partially or totally protect these individuals against infection by SARS-CoV-2.

**Figure 5:**
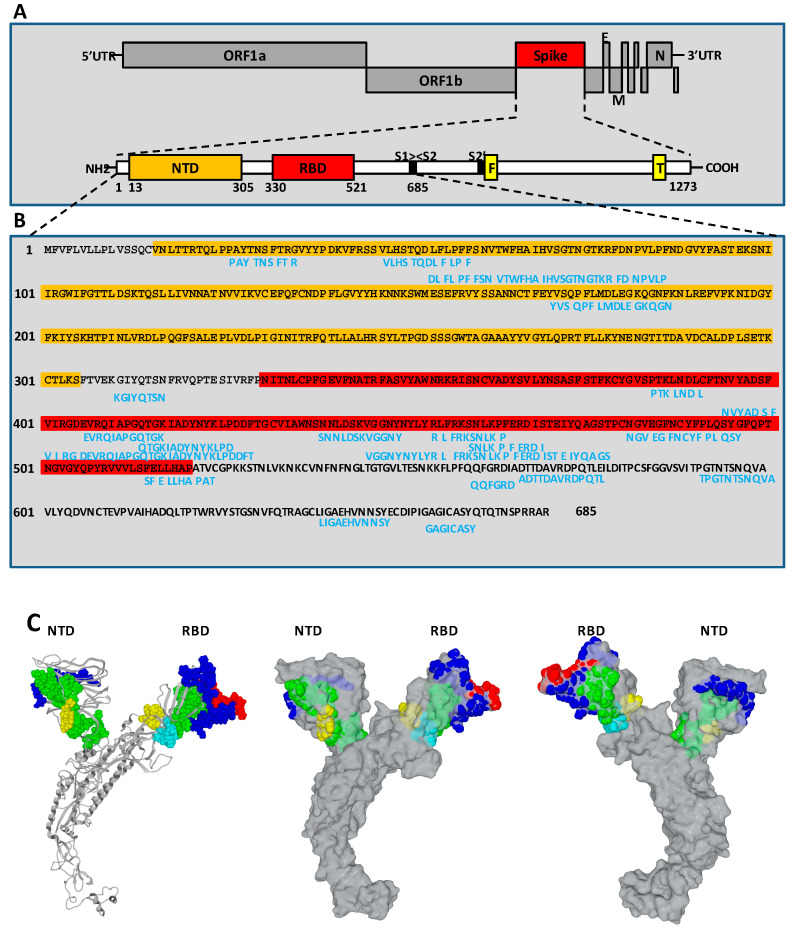
SARS-CoV-2 S1 linear epitopes. (**A**) Schematic description of the genome of SARS-CoV-2 including the 5′UTR, ORF1a and 1b that encodes 16 non-structural proteins, four structural genes encoding the S, M and E proteins that cover the virus and the N protein and six other genes encoding ORF3a, 6, 7a, 7b, 8, 10 accessory proteins at the 3′-UTR end (upper panel). Schematic representation of the SARS-CoV-2 spike protein domains (lower panel). The spike (S) protein is comprised of an N-terminal subunit (S1) that mediates the receptor binding and a C-terminal subunit (S2) responsible for virus-cell membrane fusion. NTD: N-terminal domain; RBD: receptor-binding domain; F: fusion peptide; T: single-span transmembrane domain. (**B**) Sequence of the SARS-CoV-2 spike S1 protein (amino acids 1 to 685 region) (Ref: UniProtKB/Swiss-Prot:P0DTC2.1). The main predicted SARS-CoV-2 spike S1 linear B cell epitopes are shown in blues (for details see Ahmed and colleagues [48], who identified 23 linear B cell epitopes from S, of which 20 are located in subunit S2; Shrock and colleagues [49], who identified 10 linear B cell epitopes from S; Yarmakovich and colleagues [100], who identified 12 linear B cell epitopes from S, of which five are located in subunit S2; Jaago and colleagues [59] who identified 15 linear B cell epitopes from S, of which five are located in subunit S2). (**C**) Localization of epitopes in the NTD and RBD of the SARS-CoV-2 spike protein. Left panel: Epitopes (colored atomic spheres) superimposed on the secondary structure of the spike protein. Middle and right panels, two opposite views of the Spike protein in surface rendition, with the epitopes represented as colored atomic spheres. Color code used for the NTD (position in the amino acid sequence): yellow, 26–34; green, 47–85 (overlapped epitopes 47–58, 53–85); and blue, 170–185. For the RBD: yellow, 384–390; green, 394–430 (overlapped epitopes 394–400, 401–430, 406–417, 414–427); blue, 438–477 (overlapped epitopes 438–449; 445–477; 454–463; 459–468); red, 481–495; and cyan, 514–523.

Regarding SARS-CoV-1, Zhu and colleagues [97] reported the characterization of two human monoclonal antibodies, m396 and S230.15, which neutralize SARS-CoV-1 by competing with ACE2, binding to the RBD of the SARS-CoV-1. Two putative hot-spot residues in the RBD (amino acids I_489_ and Y_491_) were identified within the SARS-CoV-1 spike that are likely to contribute to most of the m396-binding energy. Residues I_489_ and Y_491_ are highly conserved within the SARS-CoV-1 spike, indicating a possible mechanism of the m396 cross-reactivity. More recently, Ahmed and colleagues [48] listed 23 linear B cell epitopes from S, of which three are located in S1 and 20 are located in S2, that are shared by SARS-CoV-1 and SARS-CoV-2. They also reported on three discontinuous B cell epitopes within the S1 RBD region of SARS-CoV-1 characterized as the target for S230, m396, and 80R antibodies. Finally, they identified 22 linear epitopes shared by SARS-CoV-1 and SARS-CoV-2 in the N protein. Notably, a SARS-CoV-1 RBD-specific human neutralizing monoclonal antibody (mAb), CR3022, binds SARS-CoV-2 RBD with high affinity in a region that does not overlap with the ACE2-binding site, while other anti-SARS-CoV-1 mAbs (e.g., m396, CR3014) that target the ACE2 binding site of SARS-CoV-1 failed to bind SARS-CoV-2 [98]. SARS-CoV-1 RBD-specific polyclonal antibodies cross-reacted with the SARS-CoV-2 RBD protein and cross-neutralized SARS-CoV-2 infection [99]. An in silico study reported that the 33-mer _445_-VGGNYNYLYRLFRKSNLKPFERDISTEIYQAGS derived from the S protein at position 445 had the highest epitope score based on combined linear and conformational B cell epitope scoring, while the 33-mer _394_-NVYADSFVIRGDEVRQIAPGQTGKIADYNYKLP ranked second [100]. An elegant study by Shrock and colleagues [49] mapped several epitopes in the SARS-CoV-2 RBD including epitopes overlapping the ACE2 binding sites, revealing some of the likely binding sites for neutralizing antibodies. For example, S _414_-QTGKIADYNYKLPD (labeled E2) spans residue K_417_ in the RBD; K_417_ makes a direct contact with the human ACE2 protein in structures of ACE2 bound to the RBD. Thus, antibodies that recognize E2 are likely to block ACE2 binding and have neutralizing activity. Epitope S _454_-RLFRKSNLKP (labeled E6) also overlaps ACE2 contact residues and partially overlaps the binding site of the neutralizing antibody CB6, which suggests that antibodies recognizing this epitope also have neutralizing potential. Several other epitopes, such as S _438_-SNNLDSKVGGNY (labeled E5), also span or are adjacent to critical residues contacted by ACE2 (Figure 5).

## 5. Repeated Intra- and Interspecies Transmission of SARS-CoV-2 and the Risk of Reintroducing to Humans Variants Which Are Less Susceptible to Neutralization

Notably, HCoV-NL63, SARS-CoV-1, and SARS-CoV-2 spike proteins bind human ACE2 (hACE2), indicating that several members of the coronavirus family have developed a preferential tropism for this receptor to enter target cells [101,102].

Evidence for human-to-human transmission of SARS-CoV-2 was rapidly reported [12,103]. Although there is a debate as to whether the emergence of SARS-CoV-2 resulted from a spillover mechanism [104,105] or a circulation mechanism [106], it is well established that SARS-CoV-2 can be transmitted from humans to a large number of animal species which express an ACE2 compatible with infection [107,108,109,110]. However, due to the fact that SARS-CoV-2 evolves through a quasi-species mechanism [111,112,113,114], the intra- and interspecies polymorphism of ACE2 could also impact the immune responses of SARS-CoV-2. It has been hypothesized that there is a potential for spillback reservoir hosts to accelerate the evolution of SARS-CoV-2 [115]. The study of this interspecies circulation of SARS-CoV-2 led us [116] to recently propose a model we named the “boomerang effect” which postulates that ACE2 should be considered a force of positive selection promoting mutations in the viral spike and allowing for greater affinity of the spike for the ACE2 of the infected species. This was clearly demonstrated with the discovery of massive SARS-CoV-2 infections in farmed mink by transfer of the virus from humans to animals, followed by reinfection of humans by the virus harboring a variant spike with a mink signature [35,117,118]. A very similar scenario was described after the discovery of SARS-CoV-2-infected hamsters in Hong Kong [119]. In the two cases, the SARS-CoV-2 originating from humans and spreading to minks or hamsters were subject to ACE2-driven positive selection forced by structural constraints at the level of viral spike/ACE2 interaction. In terms of binding to human ACE2, the sub-lineage issued from the mink ACE2 selection was considered to be equivalent to the parental lineage, while the sub-lineage issued from the hamster ACE2 selection was considered to have better affinity for the human ACE2 [120,121]. Another example is the transmission of SARS-CoV-2 from human to deer and from deer to human. A pioneer work demonstrated that white-tailed deer (WTD), the predominant cervids in North America, are highly susceptible to SARS-CoV-2 (D614G variant) infection and shed high viral titers in their respiratory secretions [122]. Human-to-deer transmission events were observed [109]: SARS-CoV-2 antibodies were found in ~40% of wild WTD sampled in various states in the USA [123,124], and the detection of SARS-CoV-2 RNA in tissues and respiratory secretions collected from this species suggested recent infections [125]. Moreover, virus circulation in WTD (Odocoileus virginianus) and other cervids, including Elaphurus davidianus, Rangifer tarandus, and Odocoileus hemionus, was evidenced [126]. An observational surveillance study of SARS-CoV-2 circulation in deer in Ontario, Canada enabled identification of a highly divergent lineage of SARS-CoV-2 in WTD (B.1.641), with 76 mutations including 37 previously associated with non-human mammalian hosts, suggesting sustained evolution of SARS-CoV-2 in deer and deer-to-human transmission [127]. Although currently low, the risk of transmission to humans of new lineages is subject to increased surveillance in the USA, Canada and the UK (https://www.gov.uk/government/publications/hairs-risk-assessment-sars-cov-2-in-uk-cervid-populations/sars-cov-2-in-uk-cervid-populations-risk-to-humans, accessed on 3 May 2023)

A similar situation might possibly exist with the VOC Omicron B.1.1.529 lineage. The spike protein of this lineage contained 45 point mutations compared with the B1.1 lineage, including the N501Y substitution located at the interface of ACE2 and the spike protein RBD needed for adaptation to the murine ACE-2 [128]. Today, the Omicron lineage (BA.1) and its subvariants (the XBB and XBB.1 subvariants of SARS-CoV-2 Omicron BA.2 and the BQ.1 and BQ.1.1 subvariants of BA.5) have surpassed all other SARS-CoV-2 lineages in new infections of humans. However, the major concern for the biomedical community is that although these sub-variants have human ACE2 binding affinities comparable to their predecessors, they exhibit a better capacity to evade the anti-spike immune response. This suggest that the substitutions introduced in the viral spike after selection of minor variants in the viral quasi-species for improving viral fitness for the protein sequence of the viral receptor, followed by the ‘boomerang effect’ reintroducing this variant into the human population, may have changed the structure of epitopes that were previously targets for neutralizing antibodies. The neutralization of BQ.1, BQ.1.1, XBB, and XBB.1 (recombination variants) by sera from vaccinated and infected persons was markedly impaired, including sera from individuals boosted with a bivalent WA1/BA.5 mRNA vaccine. Monoclonal antibodies capable of neutralizing the original Omicron variant were found largely inactive against these newer subvariants [129], although some exception may exist, as reported for the ADG20 therapeutic antibody [130]. Despite their divergent evolution, it was recently demonstrated that mutations on the RBD can exhibit convergent evolution and that Omicron variants such as BA.5 reduce the diversity of neutralizing antibody binding sites, while increasing the proportion of non-neutralizing antibody responses, with BQ.1.1.10 (BQ.1.1 + Y144del), BA.4.6.3, XBB, and CH.1.1 being the most antibody-evasive strains [131].

## 6. Prepandemic Cross Immunity to SARS-CoV-2

Unsurprisingly, due to the sequence identities or similarities of proteins between the different coronaviruses, the literature reports a large number of cases of cross-reactions between different coronaviruses and SARS-CoV-2, and it is broadly distributed across the viral proteome, including the spike protein with recognition of the spike RBD [47,50,52,57,132,133,134]. What appears less clearly is the frequency of people who had probably never been exposed to SARS-CoV-2 and who have cross-immunity to SARS-CoV-2 pre-existing their infection or vaccination. A study of 135 sera from healthy subjects collected in Gabon five years before the first case of COVID-19 revealed that 32 samples (23.7%) were reactive to the SARS-CoV-2 N protein [135]. According to the published data the frequency of cross-immunity to SARS-CoV-2 varies from 2.73% in the study by To and colleagues [52], who studied the seroprevalence in a Hong Kong multicohort before the pandemic, up to 88% in the study by Klompus and colleagues [57], who investigated cross-reaction using broadly reactive monoclonal antibodies. Ng and colleagues [50] reported that in a cohort of 350 SARS-CoV-2–uninfected individuals in the UK, the anti-SARS-CoV-2 spike protein IgG was detected in 5 out of 35 (14%) uninfected individual with a recent confirmed HCoV infection, while it was only 1 out 31 (3%) for individuals without a recent HCoV infection. In contrast, Madjdoubi and colleagues [133] reported that preexisting cross-reactivity to the SARS-CoV-2 S protein occurred in 90% of SARS-CoV-2 uninfected adults tested in Canada. A well-controlled epidemiologic survey aimed at searching for a pre-pandemic natural immunity acquired by some human populations in central and western Africa indicated that 19.2% of samples from pre-pandemic individuals in Congo DRC and 16.6% from pre-pandemic individuals in Senegal were able to bind to the SARS-CoV-2 S1 protein [135]. A cross-sectional study conducted on 288 stored plasma samples collected before COVID-19 (in 2017–2018) in Cameroon, found cross-reactive antibodies to SARS-CoV-2 in 13.5% of samples, of which 7.3% was IgG, 7.3% IgM, and 1.0% IgG/IgM [136]. Obviously, there are many possible biases, depending on the protein at the origin of cross-reaction (the N protein carries the most immunogenic cross-reacting epitopes compared to the S protein), the location of target amino acids sequence analyzed, the titer of the antibodies, the affinity of antibodies, the experimental methodology used to define cross-reactivity, the sensitivity of the assay, and the history of infections for the individuals included in the cohorts (e.g., if there is an ongoing epidemic in the target population or a recent viral outbreak). In addition, it was recently reported that antibody levels (titers) poorly reflect on the frequency of recirculating memory B cells generated following virus priming [137,138]. However, it is possible to conclude that a certain percentage of individuals, through previous exposure to circulating coronaviruses, are likely to have built B cell responses against some cross-reactive epitopes of the spike of SARS-CoV-2 before being infected with this virus. Moreover, a recent paper (not peer reviewed) reported the presence of pre-existing SARS-CoV-2 cross-reactive CD4+ and/or CD8+ T-cells in 89.7% of stored peripheral blood mononuclear cells (PBMC) from Ugandans collected from 2015–2017, prior to the COVID-19 pandemic [139].

## 7. Discussion

At the start of the COVID-19 pandemic, it seemed reasonable to suspect an immune cross-reaction between the commonly circulating coronaviruses and SARS-CoV-2 as a means of explaining the low number of severe COVID-19 cases in children. In this review, we have tried to question the existence of immune cross-reactions between the proteins of SARS-CoV-2 and those of other coronaviruses circulating in humans or animals to determine whether previous exposure to coronaviruses other than SARS-CoV-2 can influence the susceptibility of human populations to SARS-CoV-2 infection and the severity of COVID-19 disease.

In the case of cross-immunity, what remains essential is the ability of this cross-immunity to neutralize SARS-CoV-2 and confer protection against this virus. The characterization of the historical evolution of HCoV-229E over a period of 20 years indicated that sera capable of neutralizing HCoV-229E circulating at T = 0 decreases in neutralizing titer when tested against the “new” (T = 20 years) HCoV-229E, due to genetic drift of the viral spike amino acids sequence [140]. However, the monoclonal antibody 28D9, which targets the stem helix in the spike S2 fusion subunit (_1146_-IDFQDELDEFFKNVS) and prevents the membrane fusion function of S2 of MERS-CoV, was found to cross-react with different coronavirus including HCoV-OC43, SARS-CoV-1, and SARS-CoV-2 [141]. Recently, a human antibody named 76E1 that binds an epitope of the SARS-CoV-2 spike that comprises the highly conserved S2′ site and the fusion peptide (_809_-PSKPSKRSFIEDLLFNKVTLADAGF) showed neutralizing activity against multiple alpha- and beta-coronaviruses, possibly by blocking S2′ cleavage [142]. It was reported that the LY-CoV555 neutralizing antibody that binds the SARS-CoV-2 S1-RBD protects non-human primates against SARS-CoV-2 infection, and its half-life was estimated at 13 days [143]. However, it remains difficult to determine what level of neutralizing antibodies is needed to fight off infection. Although cross-protection from seasonal HCoV infection is still controversial, it has been reported that antibodies induced by the mRNA-1273 (Moderna vaccine) vaccination displayed a boost in neutralizing activity against HCoV-NL63, whereas AZD1222 vaccination (Astrazeneca vaccine) increased antibody neutralization against HCoV-229E [43]. This corroborates previous data obtained using the Ad26.COV2.S vaccine (Janssen vaccine), describing cross-reactivity between SARS-CoV-2 and HCoV [144]. Notably, it has been reported that patients in the USA with a recent documented infection with an endemic HCoV had a less severe COVID-19 illness [145]. The different impact of the IgM (early response), IgG (acute response), and IgA (mucosal immunity) subclasses of immunoglobulins should also be considered [146]. In addition, in this paper we focus our review on the B cell immune response, but a full picture of SARS-CoV-2 immunity is likely to extend beyond antibodies. It cannot be ignored that cross-reactive memory CD4+ T cells may be involved in protection against SARS-CoV-2, and such pre-existing cross-reactivity against SARS-CoV-2 has been detected in 20% to 50% of people who have not been exposed to SARS-CoV-2 [100,147,148,149,150].

To determine how cross-reactivity with other coronaviruses can shape anti-SARS-CoV-2 immune response, another major question is related to the duration of this cross-reactive immunity. The investigation of the late winter outbreaks of HCoV-OC43 in Michigan USA in 1966 to 1969 found that, depending on the age group, between 13% and 22% had B cell responses against the virus, with a period of three years between the HCoV-OC43 outbreaks [151]. Periods of high incidence of HCoV-229E infections have also been reported to recur on a two- or three-year cycle [152]. In Hong Kong, it was found that HCoV-NL63 infection causes more than 0.2% of hospital admissions each year in children under six years of age [153]. In the United Kingdom, a large-scale comprehensive screening for all four HCoVs via analysis of 11,661 diagnostic respiratory samples collected in Edinburgh over a three-year period (between July 2006 and June 2009) using multiplex real-time reverse transcription-PCR (RT-PCR) assays, found HCoVs in between 0.3% and 0.85% of samples in all age groups [8]. A study aimed at defining the incidence and clinical features of upper respiratory infections (URI) and lower respiratory infection (LRI) associated with HCoV-NL63, HCoV-OC43, and HCoV-229E, in a cohort of otherwise healthy children below 5 years of age followed prospectively during multiple respiratory seasons, found an incidence of HCoV associated URI of 3%, and 4.8% for the HCoV-associated LRI [154]. A more recent review indicated that common circulating HCoVs can be isolated from 4% to 6% of children hospitalized for acute respiratory tract infections and from 8% of children in outpatient settings, and that HCoV-NL63 and HCoV-OC43 were the most frequent isolated species of the four common circulating HCoVs [155]. Moreover, in 10–45% of cases, common circulating HCoVs are found as co-infections with other respiratory viruses. In an epidemiologic study in adults, HCoVs were estimated to cause about 15% of adult common colds. Coronaviruses were found to cause epidemics every two to three years, with reinfections being common [156]. These HCoVs spread in humans in the Northern hemisphere between December and May, and in the Southern hemisphere between March and November (with peaks in late winter/early spring for HCoV-229E and HCoV-OC43 and in autumn for HCoV-NL63), while HCoV-HKU1 has been reported to mainly spread in the spring and summer in Asia, but in the winter and spring in the United Kingdom and South America. More recently, in the study by Sagar and colleagues [145], 875 out of 15,928 individuals (about 5.5%) were found to be positive for an endemic HCoV [145]. This suggests that the probability for a population with such a modest level of priming by HCoV to benefit from cross-reactions in the event of infection with SARS-CoV-2 is relatively low. This could only be conceivable in the event that a high percentage of the population would be reached, conferring collective immunity (at least 30% or more). Several articles document this subject. One study aimed at documenting the clinical impact of HCoV-229E and HCoV-OC43 over four consecutive winters (1999–2003) in Rochester, USA indicated that the annual infection rates ranged from 2.8% to 26% in prospective cohorts (398 HCoV infections identified), and prevalence ranged from 3.3% to 11.1% in the hospitalized cohort [157]. In a few cases, reinfections with endemic HCoV occurred as early as six months after the primary infection with HCoV-229E and HCoV-OC43 and nine months with HCoV-NL63, without identification of variations in the strains which could explain susceptibility to reinfection, while reinfections occurred most frequently at 12 months after infection, indicating that protective immunity is short-lived [158]. In contrast, another study estimated that the average duration between infection and return to susceptibility for seasonal HCoV is between 4.4 years and 7.8 years [159]. A longitudinal study in Kenya highlighted that, of a total of 497 first infections with HCoV-OC-43, HCoV-NL63, and HCo-229E, 19.9% were re-infected at least once with the homologous virus, and most commonly (24.5%) for HCoV-NL63 within a year [160]. One major bias is related to the intensity of annual HCoV attack rates and the fact that HCoV attacks are seasonal. Among the other biases, most of these studies contained small numbers of people and included those with symptomatic disease only, suggesting that the prevalence of infections may be underestimated. Children under 5 years were found to have the highest prevalence of infection with β-HCoV [8,161], possibly related to higher titers of neutralizing anti-SARS-CoV-2 cross-reactive antibodies and a decrease in infection rates and disease severity [36,50,162]. However, this remains debated, and one model concludes that cross-protection from previous HCoV infections is not sufficient to explain age differences in the incidence of SARS-CoV-2 infections but may account for some reduced susceptibility in children [159].

In 2020, despite drastic containment measures, SARS-CoV-2 rapidly spread in Asia and Europe, and many studies predicted a dramatic epidemic in Africa similar to that developing in Europe and the USA at that time. However, data did not confirm these predictions. We proposed several hypotheses that could possibly account for a late emergence and lower spread of COVID-19 in African countries, including the lack of detection and reporting of COVID-19 cases, social distancing, reduced international air traffic flows, climate, the relatively young (asymptomatic cases) and rural population, the genetic polymorphism of ACE2 or other genes involved in the control of viral replication, the use of anti-malarial drugs, and, ultimately, cross-immunity conferred by other viruses circulating in Africa [163]. Subsequently, our whole genome sequencing analyzes and those of other teams showed that African populations are sensitive to the same lineages as people from other continents [164,165,166]. Several studies have shown immune reactivity to SARS-CoV-2 proteins in samples from individuals that were collected before the COVID-19 pandemic, providing definitive evidence that SARS-CoV-2 cross-reactive immune responses may be derived from pre-existing immunity against previous non-SARS-CoV-2 infections. The prevalence of cross-reactive immune responses is likely to vary both by geographical region, by season and by age groups. One meta-analysis suggests that the prevalence of cross-reactive immune responses ranges from 0.7% in the Philippines to 21.5% in Tunisia, with 3.7% in Ivory Coast, 4.2% in South Africa, 4.8% in Uganda, 6.5% in Gabon, 8.3% in Kenya, 8.9% in Senegal, and 13.6% in Ghana [167]. Although the hypothesis of cross-immunity with endemic HCoVs was attractive, it must be admitted that prior infection with HCoV did not protect people against SARS-CoV-2 [62,63,145]. Recently, by seeking to demonstrate pre-pandemic natural immunity against SARS-CoV-2 possibly acquired by people in central and western Africa, Souris and colleagues [134] found that sera samples collected before the emergence of COVID-19 contained antibodies (IgG) against SARS-CoV-2 S1, S1-RBD, and S2. Considering the bat BANAL-52 Sarbecovirus [96] as a model and assuming that several bat species very commonly live in close contact with humans in Africa [91,168,169], Souris and colleagues [135] hypothesized that a virus close to SARS-CoV-2, which was yet to be discovered, could have circulated in humans in Africa before 2020, leading to pre-pandemic cross-reactive immunity against SARS-CoV-2. This is a possibility to the extent that for millennia, indigenous groups that depend on wildlife for their survival were exposed to the risk of pathogens’ transmission through animal hunting and wild meat consumption. In large parts of Central Africa, wildlife remains the primary source of meat and income for millions of people living in rural areas [170]. Moreover, a Merbecovirus sequence amplified from the Sub-Saharan African bat Neoromicia capensis was found to be closely related to MERS-CoV [91]. When considering the spike gene and protein, other Merbecoviruses found in Pipistrellus hesperidus in Uganda clustered with the viruses from N. capensis [91]. Similarly, Sarbecoviruses closely related to SARS-CoV-2 are likely to be found in bats from Africa. However, there are still several possible biases in this hypothesis, since the bat Sarbecovirus remains to be discovered, the cross-reactive immunity reported has not been tested so far for neutralization of SARS-CoV-2, and there is a huge difference between in vitro neutralization of a virus and in vivo protection of humans against this virus. There are still teams that seek to identify a variant-proof strategy for the development of a pan-betacoronaviruses vaccine formulation [38,171,172,173]. Additional hypotheses refer to as cross-reaction between dengue virus (DENV) antibodies and the SARS-CoV-2 S1 RBD [174]. Regarding pre-pandemic natural immunity against SARS-CoV-2, further investigations in Africa are required to determine if there could be an exception on this continent, on which contact with wild animals (and therefore the risk of infection by animal viruses) is more frequent than elsewhere, possibly contributing to curbing the pandemic.

When we analyze the extent of the COVID-19 pandemic over three years, it is clear that even if humans are frequently infected by endemic HCoV and the existence of cross-reactions may have contributed to reduce the infection by SARS-CoV-2 or severity of symptoms in some people (which however remains to be confirmed with more studies), this did not prevent the spread of the SARS-CoV-2 in the human population. In addition, although antigenic cross-reactions between SARS-CoV-2 and animal coronaviruses have been well documented, there is thus far no experimental evidence that humans can be infected with such viruses, and that, after their priming to respond to such unknown animal viruses, their immune system is able to neutralize SARS-CoV-2 and to confer a protective immunity against SARS-CoV-2. As indicated earlier in this manuscript, the duration of immunity to coronaviruses is known to be quite short. The short-lived immunity to HCoVs and other coronaviruses was also confirmed with SARS-CoV-2, for which reinfection was commonplace [175]. In addition, one study suggested that 36% of COVID-recovered individuals are serologically non-responders [176], while another study reported evidence of natural immunity against SARS-CoV-2 spike RBD in unvaccinated US adults up to 20 months after a confirmed COVID-19 infection [177]. There was an estimated 18% probability of reinfection at about 270 days after primary infection and 34% probability of reinfection at about 450 days after the primary infection [178]. With the emergence of Omicron subvariants BA4 and BA.5, COVID immunity may have been reduced from three months to one month [179]. It was claimed that individuals who received three doses of an mRNA vaccine showed an increase in RBD-specific memory B cells, which contribute to protecting against more serious consequences of Omicron infection [180]. One recent study reported that the risk of Omicron infection was reduced by 20% to 40% after a history of vaccination or SARS-CoV-2, but that it does not eliminate transmission [181]. Overall, there is evidence to suggest that pre-existing SARS-CoV-2 cross-reactive antibodies form part of the immune response to SARS-CoV-2, alongside the de novo response [172]. However, as the levels of pre-existing antibodies are low, they are largely inconsequential in determining the clinical outcomes of SARS-CoV-2 infection.

## 8. Conclusions

Taken as a whole, the data from the literature confirm the existence of cross-reactive B cell epitopes between coronaviruses circulating in different species, including SARS-CoV-2. The cross-reactive anti-spike antibodies largely target the more conserved S2 subunit on the S protein. Sometimes these cross-reactions can involve S1 and S2 epitopes that are targets for neutralizing antibodies. At the level of a single individual recently exposed to circulating coronaviruses, it remains possible that pre-existing immunity against HCoV or other CoV could have altered his/her susceptibility to SARS-CoV-2 and/or conferred a clinical advantage against SARS-CoV-2. However, at the level of human populations, the possible existence of a prepandemic cross-immunity to SARS-CoV-2 has apparently not contributed to curbing the pandemic. Moreover, it is unclear whether, on the scale of a population exposed to an emerging pandemic virus, this antibody cross-reactivity may confer any clinical benefits to individuals (e.g., modulation of the severity of SARS-CoV-2 infection). The short duration of B cells immunity, as well as both epidemiological and clinical data, suggests that the lack of pre-existing sterilizing immunity is likely to be one reason for the fast rampant spread of SARS-CoV-2 and that the existence of cross-reactions with other human and/or animal coronaviruses did not play a major role on the SARS-CoV-2 pandemic in most countries. Whether that was different in Africa remains speculative and should be analyzed further. Moreover, the impact of a recent pre-existing immunity against seasonal human coronavirus (HCoV) on the severity of COVID-19 at an individual level remains to be documented further. Finally, this study underlines the fact that we cannot limit ourselves to the quantification of anti-spike antibodies as a predictive biomarker of the outcome of COVID-19, and that vaccine-based induction of a protective immunity against SARS-CoV-2 is likely to require recognition of multiple proteins, inducing memory B and T cell responses.

## Data Availability

Not applicable.
